# Does the “state of disaster” response have a downside? Hospital incident command group leaders’ experiences of a terrorist-induced major incident: a qualitative study

**DOI:** 10.1186/s12873-025-01173-4

**Published:** 2025-02-04

**Authors:** Jason P. Murphy, Anna Hörberg, Monica Rådestad RN, Lisa Kurland, Maria Jirwe

**Affiliations:** 1 Department of Diagnostics and Intervention, Umeå University, Umeå, Sweden; 2 School of Health and Welfare, Dalarna University, Falun, Sweden; 3 Department of Clinical Science and Education, Karolinska Institutet, Stockholm, Sweden; 4 Capio S:t Göran’s hospital, Region Stockholm, Region Stockholm, Sweden; 5 Department of Medical Sciences, Örebro University, Örebro, Sweden; 6 Institution for Health Sciences, Swedish Red Cross University, Hälsovägen 11C, 141 57, Huddinge, Sweden

**Keywords:** Hospital incident command, Disaster medicine, Disaster preparedness, Decision-making, Major incident

## Abstract

**Aim:**

This study explores HICGs’ experience of disaster response during a terrorist-induced major incident major incident.

**Design:**

A qualitative descriptive design with individual semi-structured interviews was used.

**Methods:**

This was a qualitative study based on seven individual interviews. Participants were members of hospital incident command groups during a terror attack. The interviews were transcribed verbatim and analyzed using deductive content analysis. The SRQR checklist was used to report the findings.

**Results:**

The data created from the interviews identified barriers and facilitators for hospital response as well as aligned with previously established categories: *Expectations*,* prior experience*,* and uncertainty affect hospital incident command group response during a Major Incident* and three categories, (I) *Gaining situational awareness* (containing two subcategories), (II) *Transitioning to management* (containing three subcategories) and (III) *Experiences of hospital incident command group response* (containing two subcategories). In addition, the results suggest that an exaggerated response may have led to unanticipated adverse events.

**Clinical trial number:**

Not applicable.

**Supplementary Information:**

The online version contains supplementary material available at 10.1186/s12873-025-01173-4.

## Background

Major incidents (MI) such as antagonistic incidents and pandemics, continue to threaten healthcare’s ability to provide quality care to those affected [1–3]. Due to a likely influx of patients as a result of an MI, hospitals may be required to increase their ability to receive and treat the sudden surge of patients [4–6].

Hospitals are vital in mitigating morbidity and mortality caused by major incidents and are expected to maintain quality of care regardless of the type of event [7, 8]. Hospital incident command groups (HICG), commonly consisting of an incident commander and representatives from key departments within the hospital, are often activated in conjunction with an MI [9]. HICGs support hospital response through timely and appropriate decision-making concerning the allocation of resources [10]. A main responsibility of the HICG is to provide strategic leadership [9]. To do this, HICGs must possess the ability to make reliable decisions sometimes based on limited or scarce information [11].

Situational awareness is paramount for reliant decision-making yet, previous research has reported that decision-making is hampered by a lack of experience and knowledge [12]. Situational awareness is the perception and understanding of the situation the HICG faces and is vital for effective decision-making [13]. While training and simulation have been proven to be of value when the unfolding events of the MI don’t mirror training scenarios, situational awareness, and decision-making have proven difficult [14].

Uncertainty when making vital decisions is common within healthcare [15]. An overflow of unreliable information and suspected misinformation in combination with a lack of reliable information from trusted sources, impedes situational awareness. Indeed, a recent study concluded that cross-domain methodologies for detecting misinformation may facilitate decision-making [16]. Decision-making is often facilitated by reducing uncertainty and applying past experiences and training [17]. However, there is evidence that decision-making is also affected by the type of event and level of uncertainty [12] and that HICGs have made decisions to reduce uncertainty such as deciding on the highest level of hospital response when a sudden surge of patients is expected [14].

The ability of HICGs to proactively make reliable decisions has been correlated to overall response [18]. Hospital incident command groups facilitate hospital response by deciding on disaster response action levels. In the current study setting, this consists of three levels [19] with each level facilitating response by increasing competency and resources with the highest level of response, “state of disaster” which entails the automatic activation of the entire hospital and allocation of resources by cancellation of elective surgery and using reverse triage to transfer patients to other wards or home [20].

Evaluating and assessing hospital preparedness and response for actual incidents is challenging due to the infrequency of events. Previous studies have assessed HICGs’ functional capacity [21, 22]. as well as through simulations utilizing measurable indicators [9]. A previous study in the current setting, evaluated preparedness and response via a qualitative interview study with disaster preparedness coordinators (DPC) identifying that factors such as experience, expectations, and uncertainty affect HICG decision-making and indicated that various members of the HICG lacked experience and training [14]. To reduce uncertainty caused in part by the lack of reliable information, HICGs have taken measures that in in hindsight may have been exaggerated and unnecessary and may have led to unintentional, adverse events [14]. However, while DPCs are vital in disaster preparedness and response, the responsibility of guiding a hospital’s strategic response is guided by other authoritative members of the HICG. To the authors’ knowledge, this had not been explored in the current study setting and their experience as explored through in an in-depth qualitative study may provide valuable insight. This study aims to explore HICGs’ perceived experience of disaster response during an MI by applying previously identified categories for HICG disaster response.

## Method

### Design

A qualitative descriptive design (guided by the SRQR checklist) with semi-structured individual interviews was used.

### Setting and participants

On April 4th 2017, a major incident caused by the terrorist attack in Stockholm, Sweden occurred [23]. In addition to regional and local disaster plans being activated, local hospitals activated disaster plans which entailed the establishment of HICGs. A previous study concerning this event focused on disaster preparedness coordinators (DCP) experiences of HICGs’ response and preparedness [14]. The hospitals in the current study setting had held simulations in a similar theme to the MI approximately six months prior to the MI. Thus, the DPCs’ experiences of HICG response during the MI were interwoven with both experiences and expectations, based on previous training. DPCs have a comprehensive understanding of the hospital’s preparedness and structure, which affects their expectations of hospital response. Members of the HICG are expected to be well-versed in their hospital’s disaster preparedness plans.

A purposive sampling technique was used to identify participants. A DPC and co-author aided in the identification of participants meeting the inclusion criteria. The inclusion criteria were members of the HICGs holding either an analytical or a leadership role at one of the major hospitals in the study setting during the major incident caused by a terror attack [23]. A total of 13 potential participants from six hospitals were identified and seven agreed to participate, (Table 1).

**Table 1:** Description of the participants *n* = 7

Gender (male/female)	5 (71%) /2 (29%)	
Profession (physician/ RN)	6 (86%)/1(14%)	
	Mean (years)	Range (years)
Age	55	45–62
Years as a member of the HICG	8	1–17
Years in profession	25	16–32

### Data collection

Semi-structured, individual interviews were conducted in 2021 between August and September using the interview guide from the previous study [14]. The interview guide utilized in this study and based on the previous study, was developed by JM, MR, and MJ. MR was employed as a DPC during the previous simulations and terror attack and provided expert opinion concerning the type of questions to be included. JM had previously established contact with the DPCs evaluating HICG and provided expertise in the subject area while MJ provided expertise in the method of forming the questions and ensured all questions were focused on the aim of the study. In order to facilitate participation, comfort, and a sense of security for the participants, all interviews were either face-to-face (*n* = 2) at a place of their convenience, or via Zoom ^®^ (*n* = 5) based on the wishes of participants. The participants were aware of the interviewer’s research background. Care was taken to inform and motivate participation without applying unethical pressure to participate. Each interview started with an open and broad question, “It’s been four years since this incident, in which on a Friday afternoon, you received information about an antagonistic event, what do you recall?” The individual interviews ranged from 20:15–45:26 min and resulted in 245 min of interview data. The transcripts and audio files were coded and anonymized.

### Analysis

A deductive content analysis was conducted with the aim of testing by established categories [24], .All interviews were digitally recorded and transcribed verbatim. A structured categorization matrix was developed from the results of the previous study [14]. Trustworthiness was ensured by employing the analysis scheme as Elo et al., using following the three phases [25]: *In the preparation phase*, the interviews were transcribed verbatim, by JM and an overall understanding of the material was reached through multiple readings of the interviews. *In the organization phase*, the categorization matrix was initially applied to the transcribed interviews by JM, identifying similarities and differences, aided by the use of Excel ^®^. To enhance dependability, established procedures for data collection and analysis as reported in the reporting and preparation phases according to Elo et al. (2014), were adhered to. In the *reporting phase*, credibility was enhanced through the analysis procedure. The initial analysis was conducted by the first author, with the categories being discussed back and forth between the authors until consensus was reached. and *In the preparation phase*, the interviews were transcribed verbatim, and an overall understanding of the material was reached through multiple readings of the interviews.

## Results

The result of the analysis shows that the categorization matrix could be used to evaluate HICGs’ disaster response. Furthermore, this study identified barriers and facilitators to gaining situational awareness and effective decision-making (Table 2). In addition, decision-making during an MI is primarily reactive and that uncertainty may lead to excessive actions which may have unintended adverse effects.

**Table 2:** Categorization matrix based on previous results [14]

Main Category	Category	Description	Sub-category	Description
**Expectations**,** previous experience**,** and uncertainty affect HICG response during a Major Incident**	**Gaining situational awareness**	What was the process for gaining situational awareness?	Deciphering the overflow of information	How did the participants handle the initial flow of information?
			Activating and forming the HICG	What were the factors affecting the activation and formation of the HICG?
	**Transitioning to management**	What were the challenges or facilitators for transitioning to management?	Managing staff overflow	How did the surge of staff affect transitioning to management?
			Struggling with Staff briefings	What factors affected staff briefings?
			Managing the information vacuum	How did the lack of information from trusted sources affect the transition to management?
	**Actions taken during uncertainty**	How did uncertainty affect decision-making and actions taken?	An excessive response due to uncertainty	What factors affected hospital response?
			Experience of balancing staff	What were the experiences of managing staff?

### Expectations, previous experience, and uncertainty affect HICG response during a major incident

Several factors affected HICG response. While external factors beyond the realm of HICG were present, expectations, experience, and uncertainty were the main factors affecting hospital response with uncertainty permeating response. The following categories described the factors affecting HICGs’ ability to lead hospital response. The categories identify both facilitators and barriers to hospital response.

### Gaining situational awareness

This category describes efforts to form the HICG in gaining situational awareness. Grasping the breadth of the incident and the potential somatic consequences to make timely and adequate decisions, was a challenge for the HICG due in part to a lack of unconfirmed information. Additionally, circumstances that HICGs either had not previously experienced or trained for, as well as unfamiliarity with rarely used routines exacerbated uncertainty and negatively affected the formation of HICGs. Due to the lack of communication with official sources, HICGs also attempted to use the multitude of unofficial sources to monitor events as they unfolded, piecing the different varying reports in an attempt to gain situational awareness.

#### Deciphering the overflow of information

Members of the HICG described factors affecting decision-making and situational awareness. A recurring challenge was the overflow of unconfirmed information and a concern for disinformation. Initially, information of the incident was delivered through multiple sources such as media and social media. However, the overflow of information from these sources was seen as a barrier to acquiring adequate situational awareness due to an inability to confirm the information. Furthermore, members of the HICG described the overflow of information from media sources as conflictual y to official sources. These factors, in conjunction with a perceived lack of contact with the official sources of information, caused uncertainty and prohibited proactive decision-making.

*#1 ” The first information came through mass media*,* the TV. We called the regional incident command… there was a lot of information*,* and we were initially confused with unconfirmed information about reported gunfire…. we only knew that this was for real.*

#### Activating and forming the HICG

Activation of the HICG was paramount. Factors initially impeding full mobilization of the HICG were inexperience, expectations as well as insecurity and uncertainty, exacerbated for extenuating circumstances that HICGs weren’t expecting. In addition to the uncertainty, the overflow of information hampered the formation of the HICG by circumstances outside of their control and experience. While the timing of this particular MI was seen as favorable due to the amount of personnel at the hospital, certain functions were missing due to difficulties getting to the hospital due to the shutdown of public transportation as decided by city officials.

*#3 “As luck would have it*,* it was Friday*,* the morning personnel were still there*,* the evening personnel were there and management was at the hospital*,* they hadn’t yet gone home”*.

*#7 “What surprised me*,* something we hadn’t seen or trained for*,* was the decision to shut down public transportation…it made it difficult to both send patients home and get personnel in… that was something we could have trained.”*

Another factor affecting activation of the HICG was unfamiliarity with routines, such as notifying certain members of the HICG.

*#3 “there were a few that hadn’t been notified*,* due to routines not being strictly adhered to*,* and that was a problem*,* for example*,* the disaster preparedness coordinator.”*

Despite factors negatively impacting the formation and activation of the HICG, HICGs were formed rather quickly, often within 15–30 min of the incident commander receiving notification.

*# 4 “It was a little like starting an old Volvo. Initially it wasn’t working well at all but after a little while*,* it worked better and better*,* this old Volvo.”*

### Transitioning to management

The ability to transition from forming the HICG to management and decision-making is dependent upon the ability to acquire situational awareness. While the information overflow was seen as a challenge, including rumors of further incidents; also, the lack of confirmed information from official sources was seen as a barrier to gaining situational awareness.

#### Managing staff overflow

The date and time of the incident was primarily seen as advantageous as personnel from both the day and evening shifts were still at the hospital. Initially, the large number of staff gave a sense of security. However, the surge of volunteers was also seen as a challenge for effective management.

*#5 “There were a lot of people that offered to help. There were many colleagues calling my private telephone. There were so many calls that I had to find a secretary to handle the calls.”*

While some members of the HICG had difficulties either being notified or arriving to the hospital, the overflow of staff was also seen as a barrier for effective management.

*#6 “There were too many people in the room. There were a few older members nearing retirement that wanted to be there*,* to help.”*

#### Struggling with staff briefings

HICGs are often trained to hold frequent staff briefings. While the majority of HICGs were functional rather quickly and the majority held frequent briefings, several factors affected staff briefings. Uncertainty, inexperience, and the surge of personnel were factors that negatively impacted staff briefings.

#4 *It was the lack of information that made it difficult in the beginning.*

While some HICGs experienced a lack of staff briefings correlated to a lack of experience at the leadership level, other HICGs compensated initially with too many staff briefings, according to the participants.

#6 “*Initially*,* we had staff briefings earlier than what we have learned. We had been taught not to have so many staff briefings*,* but I think it was the right thing to do because we needed to share the information we had.”*

While the information vacuum with regional command affected staff briefings, briefings were also affected by the uncertainty of the effectiveness of two-way communication with other units such as the emergency department.

*#2 “The was uncertainty concerning how well our communication with for example the emergency department worked*,* if we had the same understanding of the incident*.”

#### Managing the information vacuum

the participants experienced uncertainty when the expected two-way communication with previously established credible sources they’d been trained with, was initially absent. HICGs turned to other sources in an attempt to gain situational awareness.

*#2 “We watched as many sources as we could get…we followed media reports continuously and had a screen with live broadcasts*,* so we followed as many channels as we could.”*

In addition, HICGs deviated from disaster plans to compensate for the lack of confirmed information by moving the HICG to the emergency apartment.

#1 “*We quickly established the HICG in the emergency department because that was where all the people and nurses were initially.”*

However, a few participants felt that communication worked surprisingly well.

*#2 “No*,* I remember being positively surprised by how well it worked…. because it was something I was a bit concerned about beforehand because it’s not our normal way of communicating and there’s always a bit of concern”*.

### Actions taken during uncertainty

This category describes how the circumstances of a real-life major incident, coupled with expectations, affected decision-making and has since led to insight concerning the possible outcome of some of these decisions, specifically hospitals’ level of response. In addition, participants described how certain actions taken in the face of uncertainty may have been unnecessarily exaggerated and may have had unforeseen serious consequences.

#### An excessive response due to uncertainty

The majority of HICGs decided on the highest level of hospital response within minutes of forming the HICG, prior to acquiring adequate situational awareness. According to participants, the reason for deciding on ”state of disaster” was based primarily on uncertainty with the aim of balancing a perceived need for resources. Deciding on the “state of disaster” was made to increase surge capacity by retaining personnel and sending patients currently waiting to be treated at EDs either home or to other wards. While retaining personnel was seen as necessary at the time, the decision to intake “state of disaster” was later seen as not only unnecessary and exaggerated but may have had unexpected fatal consequences.

#7 *“Deciding on state of disaster was done in order to increase capacity by sending patients home. This was a dangerous decision and may have cost lives.”*

Many participants expressed that decision-making was reactive and that proactive decision-making was impeded by difficulties in attaining situational awareness. As per disaster plans, patients already at the ED were transferred to other non-trauma sections of the ED. This created an imbalance of resources, in particular considering the insight that many “medical patients” admitted to Eds for medical conditions were patients from the incident.

#### Experience of balancing staff

Many HICGs were able to lower the level of hospital response hours after the incident, allowing personnel to leave. However, a psychological perspective was highlighted. Several participants expressed that aspects of psychological treatment may have been neglected to a certain extent. HICGs expressed a concern that they may not have taken into account the length of time the psychological units of the HICG were active. They also speculated that a lack of experience may have led to little consideration being given to the long-term needs of staffing for psychological units.

#1” The psychological unit was that part of our disaster preparedness that was pressed, and they were active for several weeks…it escalated during the evening, night, the need for psychological care escalated for a week.

Another was concerned with how patients from the incident site without apparent somatic injuries were registered and initially treated as “normal” medical patients.

*#5” We had quite a lot of disaster-related patients*,* but they were registered as normal dizziness or regular cardiac palpitation (patients) though in reality they had been at the incident site and had developed anxiety and came in. So*,* they should have been tagged at the ED as being a part of the terror incident.”*

Concerning their perception of actual preparedness, many expressed that preparedness was at acceptable levels citing experience, training, time of day, and competence as being vital components. However, results in this study indicated that there was a perception that the status of their hospital’s level of preparedness was worse now than at the time of the incident citing a lack of training, competence, and a lack of recognition concerning the importance of the HICG citing the pandemic as a possible explanation.

*#6 ”It’s far worse now… we don’t have the preparedness we had five years ago. We should be happy that no large-scale events have occurred” “We haven’t had exercises.”*

## Discussion

### Discussion of findings

This study explored members of HICGs’ experiences of managing hospital response during an MI. Strategic decision-making is vital in mitigating the effects of an MI [14]. Several factors affect effective decision-making, such as the ability to gain situational awareness and uncertainty. Uncertainty permeated HICG response. While uncertainty is an ever-present factor during an MI [15], uncertainty is often caused by either a lack of empirical knowledge or experience [26]. Both factors were identified in this study. However, a lack of expectations was identified as a possible facilitator for decision-making. In the previous study [14], uncertainty was caused in part due to expectations based on previous training, particularly when participants had been trained to expect reliable information from established sources. In this study, a lack of expectations concerning reliable sources of information appears to have minimized the reliance on these sources and aided in actively enacting strategies for processing the overflow of information. This result stresses the importance of realistic training and simulation.

HICGs commonly attempt to reduce uncertainty through various tactics such as the reliance on past experiences and information gathering [17]. HICGs struggled with situational awareness. This was primarily due to a combination of an overflow of unconfirmed information as well as an unexpected breakdown of communication with previously established sources for information, creating a vacuum of information. While the breakdown of communication is common [27], HICGs expressed that it was unexpected. This appears to be due to a misalignment in training during which participants had been trained to expect a continuous flow of information from regional command. This led to some HICGs deviating from disaster plans to compensate the lack of information by adding a function to monitor as many sources of information as possible as well as moving the HICG command center to the ED to ensure an alignment in situational awareness. Adequate response appears to have been facilitated through the ability to apply experiences and knowledge to aid in adaptability. These actions may provide information that may lead to proactive decisions concerning medical ambition as well as to changes in disaster plans and training. However, recent research suggests that situational awareness may be enhanced through the dissemination of false or misinformation by employing cross-dimensional methodologies [16]. While promising, the presented methodologies may be time-consuming. Both training and specified functions for the dissemination of misinformation may aid in the successful implementation and operative application of such methods.

While staff overflow initially was seen as a factor negatively affecting management, it was also seen as necessary initially due to a lack of situational awareness. Moreover, the overflow of personnel may have mitigated the effects of the city’s decision to shut down mass transportation had the incident been prolonged. However, a well-known method for increasing a hospital’s surge capacity is employing reverse triage to facilitate the transfer of patients [28]. A hospital’s resources are finite and the ability to both transfer patients and increase resources may often be dependent on effective transport [29]. Regional collaboration with various transport agencies should be well-established and integrated in disaster preparedness plans to aid in effective surge capacity and to avoid possibly ineffective ad hoc solutions [29]. However, due to the timing of the incident, there were ample personnel and towards the end of the evening, most hospitals lowered their level of response, allowing personnel to go home. However, this study highlighted a lack of insight concerning the psychological effects of the incident. While the somatic sections of the hospital were at pre-incident levels within 24 h of the incident, the psychological unit was activated for weeks. In addition, this study also highlights the fact that many non-trauma patients from the incident were admitted as “medical” patients but not registered as “disaster patients”, which may have caused an imbalance in personnel resources as disaster plans were designed to primarily increase surge for trauma patients. This stands in contrast to previous studies focusing on the number of somatic patients [30]. According to participants, deciding on the “state of disaster” was an excessive and unnecessary action. This was done primarily to ensure adequate surge capacity. However, the decision was made primarily in the face of uncertainty as opposed to being evidence-based. In addition to knowledge being a tangible factor in decision-making, it has been suggested that emotional intelligence and self-efficacy may positively impact decision-making [31]. In hindsight, the “state of disaster” decision may have caused the fatality of a non-MI-related patient.

The participants in this study highlighted several factors affecting hospital response. While some expressed that their hospital preparedness was still at adequate levels, others expressed that hospital preparedness is much worse, due to a lack of training, education, and lack of priority from hospital management, in part due to the COVID-19 pandemic. There is a concern that a lack of well-trained HICGs and misaligned disaster plans may have negative, unintentional adverse effects.

### Strengths and limitations

The number of participants in this study could be seen as a limitation (*n* = 7) with six declining. The reason given for declining participation was lack of time. However, given the specificity of the major incident in which a finite number of potential participants met the inclusion criteria, over 50% of the invited participants accepted the invitation. While empirical support such as number of participants or number of interview minutes has been deemed to be relevant for assessing the validity of qualitative studies, it has been suggested that information power is more relevant for assessing qualitative interview studies using content analysis [32]. Sample size was assessed by applying the criteria of information power; study aim, sample specificity, quality of dialogue, analysis strategy, and established theory, as previously described [32]. *The study aim* was specific and narrow, allowing for a smaller sample size. *Sample specificity* was dense, meaning the participants had highly specific characteristics and expertise that were directly related to the aim. *Quality of dialogue* was determined to be strong due to a combination of the experience of the interviewer within the research field and both the level of expertise of the participants, and their ability to concisely articulate their experiences *Established theory*: Due to this being the second study focusing on this specific aim, no established theory was applied. However, the results from the previous study were used for developing the matrix. A small sample size can be vital given it adds important evidence to an area of research that lacks an established theory [32].

The specificity of the aim and the specificity of participants chosen based on the aim, the richness of dialog, and the analysis strategy indicate that information power was sufficient. However, given the specificity of the incident, and the specific inclusion criteria in combination with the number of participants, the results of this study may not be transferable to other settings. Despite this, these results provide rich, descriptive data pertaining to decision-making and hospital response to an MI and may be applicable given similar circumstances in other settings.

Biased interpretation is a risk when authors possess a preunderstanding of a subject. JM and MR conduct research on the subject. To minimize bias, a researcher without in-depth knowledge of the subject but with extensive experience in qualitative methods, MJ was included in all phases of the study. In an attempt to minimize investigator bias, investigator triangulation was used with AH and LK assessing the results.

An issue of concern was recall bias [33]. Nearly three and a half years had passed between the original interviews and the interviews conducted in the fall of 2021. While the event under investigation was a rare incident, the possibility of participants erroneously recalling details or not being able to remember certain aspects or recall bias was discussed [33]. However, there is evidence suggesting recollections, particularly from critical incidents are highly accurate [34]. This would appear to be supported by the participants’ recollection of the event, which is consistent with the study based on the same event that was much closer in time [14].

Since a new analysis was going to be based on knowledge gained in a previous study, a deductive content analysis approach was applied [35]. Elo et al.’s (2014) outline for trustworthiness [25] was utilized. In addition to assessing credibility and reliability, transferability is vital when assessing the trustworthiness of a study. The specificity of the study setting may not be transferable to other settings, rather transferability is determined by the reader. To facilitate transferability, care was taken to provide enough detail to contextualize the study setting and the results. Concerning the analysis method, the authors discussed and were prepared to employ other analytical methods if new data had been unearthed, such as inductive analysis. However, given the results based on the data in which no new categories were identified, deductive analysis was deemed sufficient and relevant for this study.

## Conclusion

Effective strategic decision-making at the HICG level during an MI is reliant on the ability to gain situational awareness. The ability to gain situational awareness is affected by experience, expectations, and uncertainty. The aim of disaster plans during a potential disaster is to do the most amount of good for the largest number of patients possible [36]. This is facilitated through various actions such as the redistribution of resources by deciding on the “state of disaster”. The results of this study indicate that taking this action to combat uncertainty, may have unintentional negative consequences. Targeted education and realistic training scenarios are vital in reducing uncertainty, facilitating decision-making, and mitigating the effects of MIs. This study adds valuable information concerning disaster preparedness and response. The clinical implications of this study include the possible reduction of avoidable adverse events that may be caused by ineffective decision-making and ad-hoc solutions through the implementation of misinformation management strategies. Furthermore, effective surge capacity may be facilitated in part through the inclusion of regional collaboration in disaster plans concerning effective and reliable transport of both patients and personnel. Future studies should include the construction and implementation of an evidence-based decision-making instrument as well as the evaluation of the instrument and overall hospital response.

## Electronic supplementary material

Below is the link to the electronic supplementary material.

Supplementary Material 1

## Data Availability

Due to the possibility of participants’ privacy being compromised, the data are not publicly available. However, the data that support the findings of this study are available on request from the corresponding author.

## Abbreviations

DCP: Disaster preparedness coordinator
HICG: Hospital incident command group
MI: Major incident
