# Supplementary material for: Does the “state of disaster” response have a downside? Hospital incident command group leaders’ experiences of a terrorist-induced major incident: a qualitative study
Source: BMC Emerg Med. 2025 Feb 4;25:21. doi: 10.1186/s12873-025-01173-4 (PMC11792314; doi:10.1186/s12873-025-01173-4)
Supplement: Supplementary file 1 — Supplementary Material 1 [file 12873_2025_1173_MOESM1_ESM.pdf]

Interview guide:

It has been 4 years since the event.... How long have you been a physician/RN? How long have you been an active member of the HICG? How many times have you been involved when the HICG was activated? How old are you?

1. Just before 3:00 PM on April 7, you received an alert that something significant had happened in the city, that it was some form of antagonistic event. My first question is, what were your thoughts?

2. In the initial phase after the alert, the state of preparedness was raised, and the hospital incident command group was activated: Do you recall how long it took for the hospital to establish a staff? Are you aware that there is a target value of 30 minutes or 60 minutes depending on the day or on-call period for establishing a staff?

3. Can you tell me about your experience of the startup, and what state of preparedness you had at that time?

4. How did you receive the notification about the incident? What alert system was used to call in HICG?

a. How was the startup managed at the command center?

b. Were all functions staffed? What is the plan for initiation with few staff members present?

5. On what indications did you deem it necessary to go into a state of disaster according to your assessment?

6. How did the HICG work with strategic decisions and actions on April 7 based on: a. Collecting data for decisions b. Translating decisions into direct tasks for staff members

7. What is your assessment regarding the risk of displacement effects of patients who sought care in the emergency department during an incident with large-scale casualties? Is this something you account for in the disaster plan?
8. The tasks for the HICG are to quickly allocate and redistribute resources to meet the urgently changing care needs. How did you perceive HICG's ability to:

   Create immediate capacity and did they have a long-term plan? f. Reassess the state of preparedness? g. Plan for management's sustainability and relief? h. Plan for personnel sustainability in the operation? i. Plan for materials/equipment? j. Is there a substitute location for the command center?

9. What is your perception of management work on April 7, 2017? Reactive/proactive? (Please give an example.)
10. What do you think about the HICG's ability to handle major incidents (today and in the past)? Do you receive the necessary training?

Communication

Clarifying questions:

11. How did communication work on April 7?
    a. Was the communication plan used?
    b. Did collaboration at the regional level work?
    c. Did the wards receive continuous and satisfactory information from the HICG?
    d. Patients and relatives (operations or elective care postponed)?
    e. Media, were times for press releases planned, etc.? f. External actors?

Staff Methodology

12. How did you perceive the information/communication from the staff manager/with the staff on April 7?

13. Staff tools should support the work in the staff. How did you perceive the staff's use of staff tools during the 2016 exercise compared to April 7? a. Situation report/Four-field analysis b. Staff orientation – clear and structured? c. Keeping a diary/log

d. Operational overview

e. Documentation of staff work

What do you think needs to be developed and in what way?

Conclusion

14. What went well and what areas have potential for improvement?

15. What would you like to develop to strengthen the management capacity at the hospital?

16. What is your assessment of the hospital's ability to receive a large number of patients seeking care in the emergency department related to an event but who had to wait due to a lack of ambulances, other transport means, or public transport (as on April 7 when everything stood still) and HICG's ability to make decisions on measures?
